# Admission blood glucose level and outcome in patients requiring venoarterial extracorporeal membrane oxygenation

**DOI:** 10.1007/s00392-021-01862-7

**Published:** 2021-05-04

**Authors:** Xavier Bemtgen, Jonathan Rilinger, Markus Jäckel, Viviane Zotzmann, Alexander Supady, Christoph Benk, Christoph Bode, Tobias Wengenmayer, Achim Lother, Dawid L. Staudacher

**Affiliations:** 1 Department of Cardiology and Angiology I (Heart Center Freiburg—Bad Krozingen), Faculty of Medicine, Medical Center—University of Freiburg, University of Freiburg, Hugstetterstrasse 55, 79106 Freiburg, Germany; 2 Department of Medicine III (Interdisciplinary Medical Intensive Care), Faculty of Medicine, Medical Center—University of Freiburg, University of Freiburg, Freiburg, Germany; 3 Heidelberg Institute of Global Health, University of Heidelberg, Heidelberg, Germany; 4 Department of Cardiovascular Surgery (University Heart Center Freiburg—Bad Krozingen), Faculty of Medicine, Medical Center—University of Freiburg, University of Freiburg, Freiburg, Germany

**Keywords:** V-A ECMO, Glucose, Survival, ECPR, Cardiogenic shock

## Abstract

**Background:**

Patients with cardiogenic shock or cardiac arrest undergoing venoarterial extracorporeal membrane oxygenation (V-A ECMO) frequently present with blood glucose levels out of normal range. The clinical relevance of such findings in the context of V-A ECMO is unknown. We therefore investigated the prognostic relevance of blood glucose at time of cannulation for V-A ECMO.

**Methods:**

We conducted a single-center retrospective registry study. All patients receiving V-A ECMO from October 2010 to January 2020 were included if blood glucose level at time of cannulation were documented. Patients were divided in five groups according to the initial blood glucose level ranging from hypoglycemic (< 80 mg/dl), normoglycemic (80–140 mg/dl), to mild (141-240 mg/dl), moderate (241–400 mg/dl), and severe (> 400 mg/dl) hyperglycemia, respectively. Clinical presentation, arterial blood gas analysis, and survival were compared between the groups.

**Results:**

392 patients met inclusion criteria. Median age was 62 years (51.5–70.0), SAPS II at admission was 54 (43.5–63.0), and 108/392 (27.6%) were female. 131/392 were discharged alive (hospital survival 33.4%). At time of cannulation, survivors had higher pH, hemoglobin, calcium, bicarbonate but lower potassium and lactate levels compared to non-survivors (all *p* < 0.01). Outcome of patients diagnosed with particularly high (> 400 mg/dl) and low (< 80 mg/dl) blood glucose at time of V-A ECMO cannulation, respectively, was worse compared to patients with normoglycemic, mildly or moderately elevated values (*p* = 0.02). Glucose was independently associated with poor outcome after adjustment for other predictors of survival and persisted in all investigated subgroups.

**Conclusion:**

Arterial blood glucose at time of V-A ECMO cannulation predicts in-hospital survival of patients with cardiac shock or after ECPR. Whether dysglycemia represents a potential therapeutic target requires further evaluation in prospective studies.

**Graphical Abstract:**

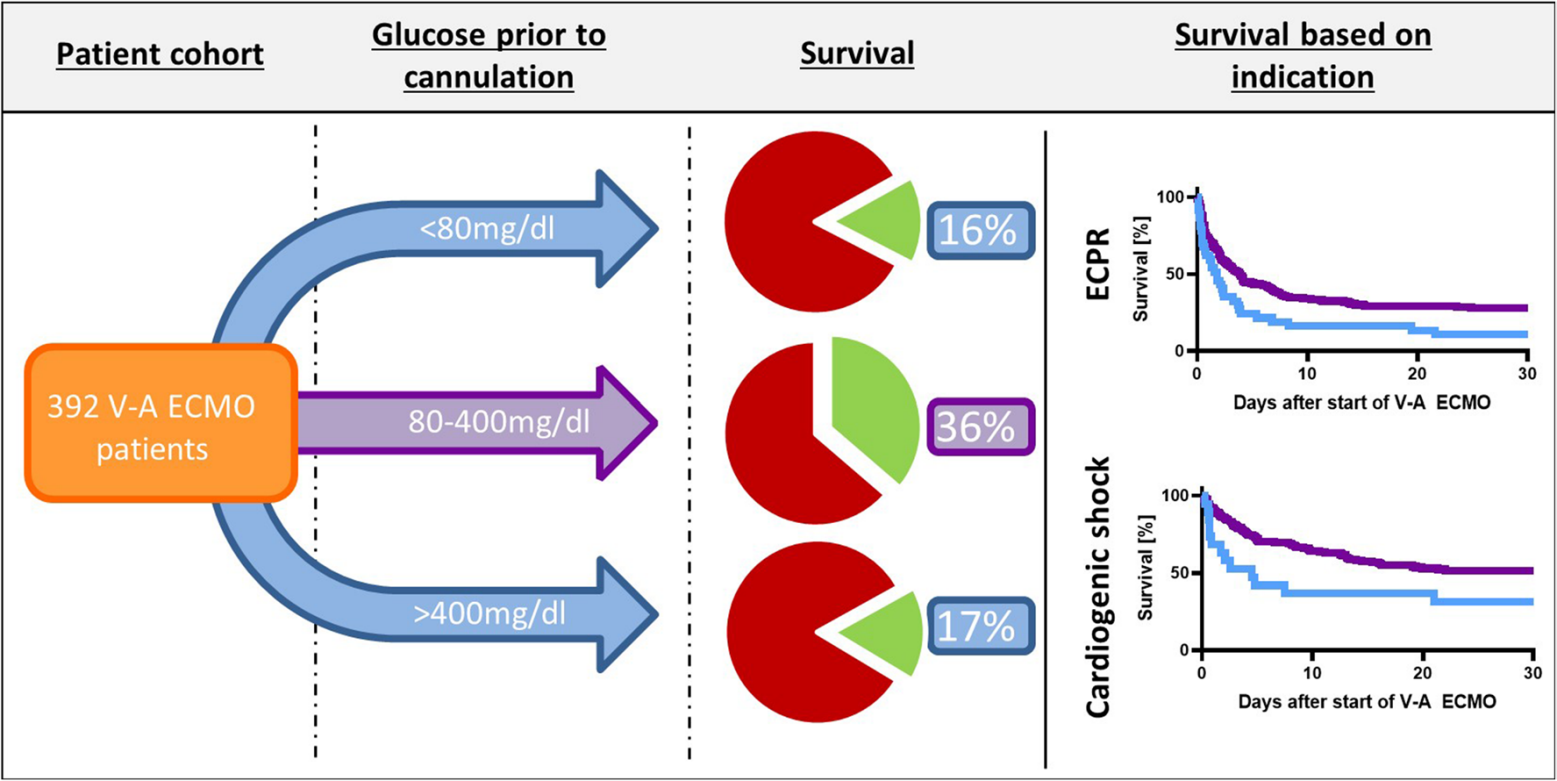

**Supplementary Information:**

The online version contains supplementary material available at 10.1007/s00392-021-01862-7.

## Background

Venoarterial extracorporeal membrane oxygenation therapy (V-A ECMO) is increasingly used for treatment of severe cardiogenic shock or for extracorporeal cardiopulmonary resuscitation (ECPR). Despite great efforts, mortality rate in patients undergoing V-A ECMO is still about 70% [1, 2], which means a great medical need for improvement.

Previously, admission blood glucose level was identified as a potential prognostic factor in patients with cardiogenic shock. Severe hypo- or hyperglycemia, respectively, was associated with increased in-hospital mortality [3]. Likewise, recent findings suggest that initial blood glucose level below 150 mg/dl is associated with adverse neurological outcome after cardiac arrest [4]. For non-diabetic patients after intra-hospital cardiac arrest (IHCA), extreme blood glucose levels during the first 24 h, hyper- and hypoglycemic, were associated with poor survival [5]. Interestingly, in a large retrospective analysis for patients after out-of-hospital cardiac arrest (OHCA), neurological outcome did not differ between treated and untreated intra-arrest hypoglycemia [6].

Several studies investigated the prognostic value of blood markers including pH, lactate, or standard bicarbonate concentration (SBC) in V-A ECMO [7, 8]. However, the impact of blood glucose has not been investigated yet. Limited data on extreme blood glucose levels only exist for pediatric patients on V-A ECMO therapy [9]. Thus, the aim of the present study was to evaluate the association between blood glucose level at time of cannulation for V-A ECMO and hospital survival.

## Methods

### Study setting

We conducted a single-center retrospective registry study. Data derive from a registry of all patients on V-A ECMO treated at a medical intensive-care unit (ICU) located at a tertiary university hospital. All patients receiving V-A ECMO from October, 2010 to January, 2020 were included in the analysis. Exclusion criteria were cannulation in the operation theatre, treatment at a different intensive-care unit and missing values for glucose level at the start of the V-A ECMO therapy. In the case of extracorporeal cardiopulmonary resuscitation (ECPR), patients after intra- as well as out-of-hospital cardiac arrest (IHCA and OHCA, respectively) were included.

### Local V-A ECMO setting

The center provides a 24/7 service for referral of patients as well as for in-house patients after and during cardiopulmonary resuscitation or in severe cardiogenic shock for V-A ECMO support. As for local policy, decision to cannulate for V-A ECMO is made after multidisciplinary discussion at the bedside. ECPR was defined as V-A ECMO cannulation during continuous cardiopulmonary resuscitation without return of spontaneous circulation (ROSC); or as V-A ECMO cannulation within the first 20 min after ROSC in case of persistent hemodynamic instability as previously suggested [10]. V-A ECMO cannulation and maintenance were done according to regularly revised standard operating procedures as described earlier [11, 12]. In brief, cannulation was performed in Seldinger’s technique by two experienced intensivists and supported by a perfusionist and additional ICU staff. ECMO systems used were SCPC (Sorin Centrifugal Pump Console, LivaNova, London, United Kingdom), Cardiohelp (Maquet Getinge Group, Rastatt, Germany), or CARL controller (Resuscitec GmbH, Freiburg, Germany). Standard venous draining cannulas were 21–23 Fr. / 55 cm and arterial return cannulas 15–17 Fr. / 23 cm (HLS cannula, Maquet Getinge Group, Rastatt, Germany). For patients without life-threatening bleeding, anticoagulation was provided by intravenous administration of unfractionated heparin aiming at a partial thromboplastin time of 50–60 s. The management of vasopressors and fluid therapy was driven by clinical judgement of the ECMO-experienced intensivist as reported earlier [13, 14].

### Blood gas analysis

Blood gas analysis was routinely made by point of care testing (POCT) and results were automatically transferred to the electronic patient files. Frequency of POCT was influenced by clinical judgement of the ICU staff, hemodynamic stability, and dynamics of the different parameters. During the first 24 h after cannulation, blood gas analyses were acquired before and after cannulation and approximately every 1–2 h afterwards. Missing values could occur because of out-of-hospital cannulation, errors during the transfer to the hospital information system, POCT machine not connected to the network or because of inaccurately entered information. A minimum of 18 different values were routinely measured with each blood gas analysis, including partial pressure of carbon dioxide (pCO_2_) and oxygen (pO_2_), hemoglobin, lactate, sodium, potassium, calcium, chloride, and base excess.

### Data acquisition and statistical analysis

Present data derived from a single-center retrospective registry analysis. Parameters at certain times during and after V-A ECMO implantation were automatically sorted and extracted from the data with a search range of ± 1 h. If no glucose value was detected at implantation, a manual search was performed. For blood gas analysis, only arterial samples were used. Patients were grouped according to their initial glucose value (< 80 mg/dl, 81–140 mg/dl, 141–240 mg/dl, 241–400 mg/dl, > 400 mg/dl), which were altered from [3]. Additionally, a second analysis was conducted separating extreme values from moderate measurements. One group consisted of extreme glucose values below 80 mg/dl and above 400 mg/dl, respectively. These patients were compared to all remaining patients with normal, mildly, or moderately elevate blood glucose (81–400 mg/dl). Apart from glucose and other laboratory parameters, we recorded patient demographics and medical history, occurrence of different events (e.g., decannulation, death, discharge from ICU) and different severity of disease scores. For data analysis, SPSS (version 23, IBM Statistics), Python programming language (version 3.8.3, Python Software Foundation), and Prism (version 8, GraphPad) were used. For statistical analysis, unpaired t test, Fisher’s exact/Chi-square test, and Log-rank/Gehan–Breslow test were used as applicable. Multivariate logistic regression analysis was performed for known predictors of survival in patients with V-A ECMO (ECPR, age, lactate, pH, glucose, female gender, and diabetes mellitus). For Cox regression analysis, independent predictors of hospital survival detected in the multivariate logistic regression analysis were included in the model. A p value of < 0.05 was considered statistically significant. All categorical variables were presented in absolute number (percent of all patients) or were presented as median with inter-quartile range for continuous variables if not stated otherwise.

## Results

**Fig. 1:**
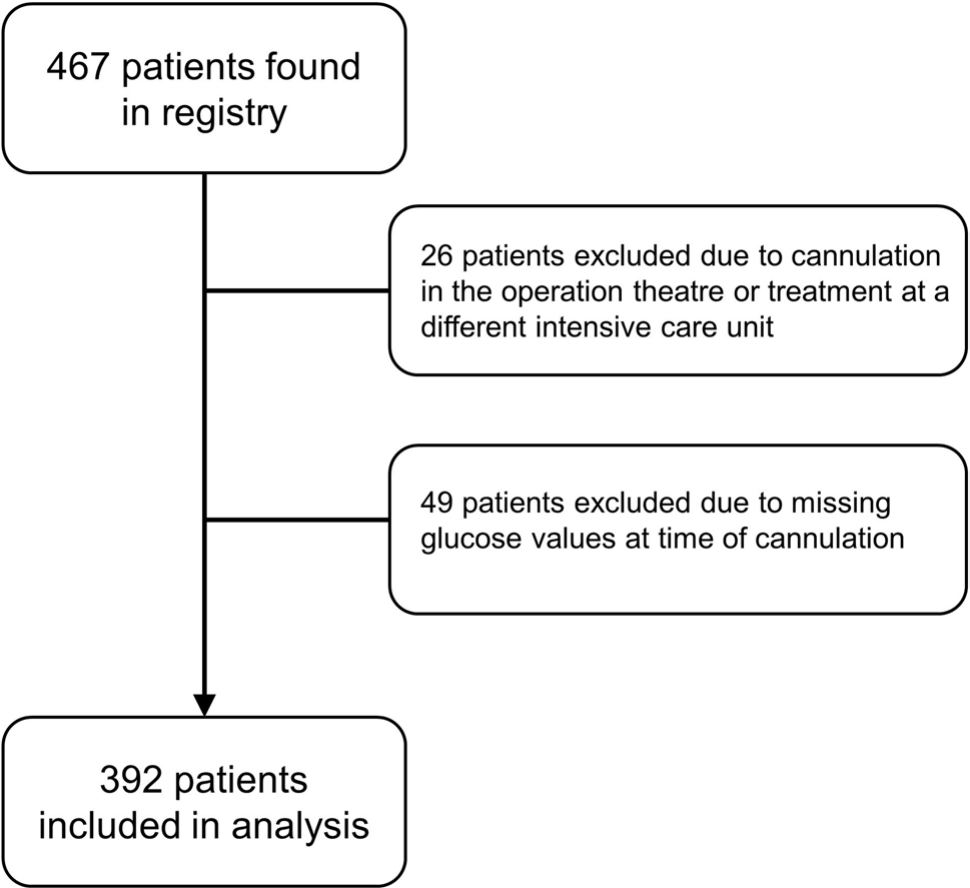
Patient selection

Patient cohort: A total of 467 V-A ECMO patients were screened and 392 met the inclusion criteria (Fig. 1). Median age was 62 years (51.5–70.0 years) at time of V-A ECMO implantation and the group consisted of 284 men and 108 women (27.6%) (Table 1). At time of initiation for V-A ECMO, 173/392 (44%) were in cardiogenic shock and 219/392 (56%) had a cardiac arrest. There was no difference in the rate of comorbidities, age, sex, and BMI between the five groups. Both dysglycemic groups (< 80 mg/dl and > 400 mg/dl) had a trend towards higher SAPS II scores at admission and lower overall duration of ICU stay and V-A ECMO duration compared to the other groups. Survival was higher in the cardiogenic shock group, and age, sex, body measurements, and severity of illness were similar between survivors and non-survivors (suppl. Table 1, online supplement). When comparing blood gas analyses for the different groups, patients with blood glucose levels < 80 mg/dl or > 400 mg/dl had a trend towards lower pH, hemoglobin, bicarbonate but higher lactate levels compared to the other groups (Table 2). At time of cannulation, survivors had higher pH, hemoglobin, calcium, bicarbonate but lower potassium and lactate levels compared to non-survivors (all *p* < 0.01, suppl. Table 2, online supplement).

**Table 1:** Patients’ characteristics and path of ICU stay

Glucose level	0–80 mg/dl (*n* = 32)	81–140 mg/dl (*n* = 89)	141–240 mg/dl (*n* = 143)	241–400 mg/dl (*n* = 104)	> 400 mg/dl (*n* = 24)	All patients (*n* = 392)
Hospital survival	5 (15.6%)	28 (31.5%)	59 (41.3%)	35 (33.7%)	4 (16.7%)	131 (33.4%)
Female sex	10 (31.6%)	28 (31.5%)	33 (23.1%)	28 (26.9%)	9 (37.5%)	108 (27.6%)
Age [years]	61.0 (42.0–69.0)	62.0 (53.0–71.0)	62.0 (49.0–71.0)	62.0 (53.0–70.5)	61.5 (50.0–70.0)	62.0 (51.5–70.0)
BMI [kg/m2]	26.2 (24.8–27.7)	26.2 (24.2–27.7)	26.36 (24.2–28.1)	26.7 (24.8–27.0)	26.7 (25.6–30.8)	26.7 (24.2–27.7)
CAD	10 (31.3%)	47 (52.8%)	80 (55.9%)	67 (64.4%)	12 (50.0%)	216 (55.1%)
Arterial hypertension	13 (40.6%)	40 (44.9%)	64 (44.8%)	35 (33.7%)	11 (45.8%)	163 (41.6%)
PAD	3 (9.4%)	7 (7.9%)	10 (7.0%)	4 (3.9%)	2 (8.3%)	26 (6.6%)
Liver disease	5 (15.6%)	8 (9.0%)	6 (4.2%)	6 (5.8%)	1 (4.2%)	26 (6.6%)
Renal disease	5 (15.6%)	13 (14.6%)	24 (16.8%)	19 (18.3%)	2 (8.3%)	63 (16.1%)
Diabetes mellitus	10 (31.3%)	17 (19.1%)	31 (21.7%)	30 (28.9%)	11 (45.8%)	99 (25.3%)
Lung disease	3 (9.4%)	23 (25.8%)	19 (13.3%)	14 (13.5%)	3 (12.5%)	62 (15.8%)
SAPS II at admission	59.5 (50–72)	55 (42–66)	52 (40–59)	54 (46.0–62.5)	59.5 (46.5–66.5)	54 (43.5–63.0)
Duration of ICU stay [days]	1.8 (0.4–3.9)	5.6 (1.6–17.1)	6.5 (2.1–14.8)	6.4 (1.5–13.5)	2.3 (1.1–8.6)	5 (1.5–13.9)
V-A ECMO duration [hours]	46.4 (10.6–90.3)	73.2 (27.5–134.4)	71.7 (26.5–117.8)	73.3 (35.6–121.9)	51.5 (19.9–105.7)	68.6 (26.3–119.7)
Indication for V-A ECMO						
Cardiogenic shock	13 (40.6%)	53 (59.6%)	74 (51.8%)	27 (26.0%)	6 (25.0%)	173 (44.1%)
ECPR	19 (59.4%)	36 (40.5%)	69 (48.3%)	77 (74.0%)	18 (75.0%)	219 (55.9%)

*BMI* Body mass index, *CAD* Coronary artery disease, *PAD* Peripheral artery disease, *SAPS* *II* Simplified Acute Physiology Score II, *ICU* Intensive-care unit, *V-A ECMO* venoarterial extracorporeal membrane oxygenation, *ECPR* extracorporeal cardiopulmonary resuscitation

**Table 2:** Laboratory and POCT values at time of cannulation for V-A ECMO

Glucose level	0–80 mg/dl (*n* = 32)	81–140 mg/dl (*n* = 89)	141–240 mg/dl (*n* = 143)	241–400 mg/dl (*n* = 104)	> 400 mg/dl (*n* = 24)	All patients (*n* = 392)
HbA1c [%]	6.2 (5.7–7.4)	5.9 (5.5–6.2)	5.6 (5.2–6.1)	6.1 (5.45–7.05)	6.7 (5.6–8.9)	5.8 (5.3–6.4)
Glucose [mg/dl]	60.0 (46–72.5)	115.0 (103.0–123.0)	182.0 (165.0–209.0)	298.5 (265.5–346.0)	445.5 (421.5–472.5)	184.5 (122.5–272.0)
pH	7.17 (7.03–7.30)	7.28 (7.19–7.39)	7.29 (7.21–7.37)	7.23 (7.12–7.32)	7.2 (7.13–7.30)	7.27 (7.17–7.35)
pCO_2_ [mmHg]	42.4 (30.1–51.1)	37.0 (31.3–43.9)	38.2 (33.1–45.5)	40.3 (34.0–47.5)	35.2 (31.6–42.9)	38.8 (32.7–45.6)
pO_2_ [mmHg]	165.0 (68.0–394.0)	152.0 (102.5–279.0)	209.0 (117.0–369.0)	168.5 (85.6–301.0)	366.0 (193.0–485.0)	184.0 (102.0–344.0)
SpO_2_ [%]	97.6 (87.1–99.7)	98.8 (95.3–99.6)	99.2 (97.9–99.7)	98.5 (95.8–99.6)	99.6 (99.0–100.0)	99.0 (97.0–99.7)
HCO_3_ [mmol/l]	14.4 (12.3–18.1)	17.1 (13.7–21.8)	18.5 (15.0–21.7)	16.8 (13.8–19.35)	13.3 (11.6–19.7)	17.2 (13.7–20.7)
SBC	13.8 (11.8–16.2)	18.0 (14.4–22.6)	18.2 (15.6–21. 5)	16.4 (13.6–19.6)	12.9 (11.0–18.6)	17.4 (13.6–21.0)
Hb [g/dl]	8.3 (6.9–11.3)	9.5 (7.4–11.6)	10.5 (8.7–12.3)	10.9 (9.0–12.6)	9.4 (7.5–11.8)	10.1 (8.2–12.2)
HCT [%]	25.1 (21.4–31.4)	29.4 (23.1–35.1)	31.3 (26.3–37.4)	32.5 (27.1–38.2)	29.4 (20.3–36.2)	30.6 (25.1–36.8)
Sodium [mmol/l]	140.1 (136.0–145.0)	141.0 (135.0–145.0)	139.0 (135.0–142.0)	139.8 (136.0–144.0)	137.4 (131.0–142.0)	139.2 (135.0–143.0)
Potassium [mmol/l]	5.0 (4.1–5.8)	4.5 (4.0–5.1)	4.3 (3.7–4.9)	4 (3.5–4. 6)	4.2 (3.6–5.0)	4.3 (3.7–5.0)
Chloride [mmol/l]	108.0 (104.5–110.5)	106.0 (102.0–110.0)	107.0 (103.0–111.0)	106.0 (102.0–109.0)	104.0 (101.0–105.0)	106.0 (102.0–110.0)
Calcium [mmol/l]	1.1 (1.0–1.1)	1.1 (1.0–1.1)	1.1 (1.0–1.2)	1.1 (1.0–1.2)	1.1 (1.0–1.2)	1.1 (1.0–1.2)
Lactate [mmol/l]	12.4 (10.0–16.0)	8.3 (3.2–12.4)	7.3 (3. 5–12.7)	10.5 (7.7–13.8)	13.4 (10.2–16.9)	9.6 (4.9–13.4)
Bilirubine [mg/dl]	1.3 (0.9–2.8)	1.1 (0.6–2.1)	0.8 (0.45–1.2)	0.6 (0.3–0.9)	0.5 (0.3–1.3)	0.8 (0.5–1.4)

*pCO*_*2*_ partial pressure of carbon dioxide, *pO*_*2*_ partial pressure of oxygen, *SpO*_*2*_ peripheral oxygen saturation, *Hb* Hemoglobin, *HCO*_*3*_ Bicarbonate, *HCT* Hematocrit, *SBC* Standard bicarbonate concentration

**Fig. 2:**
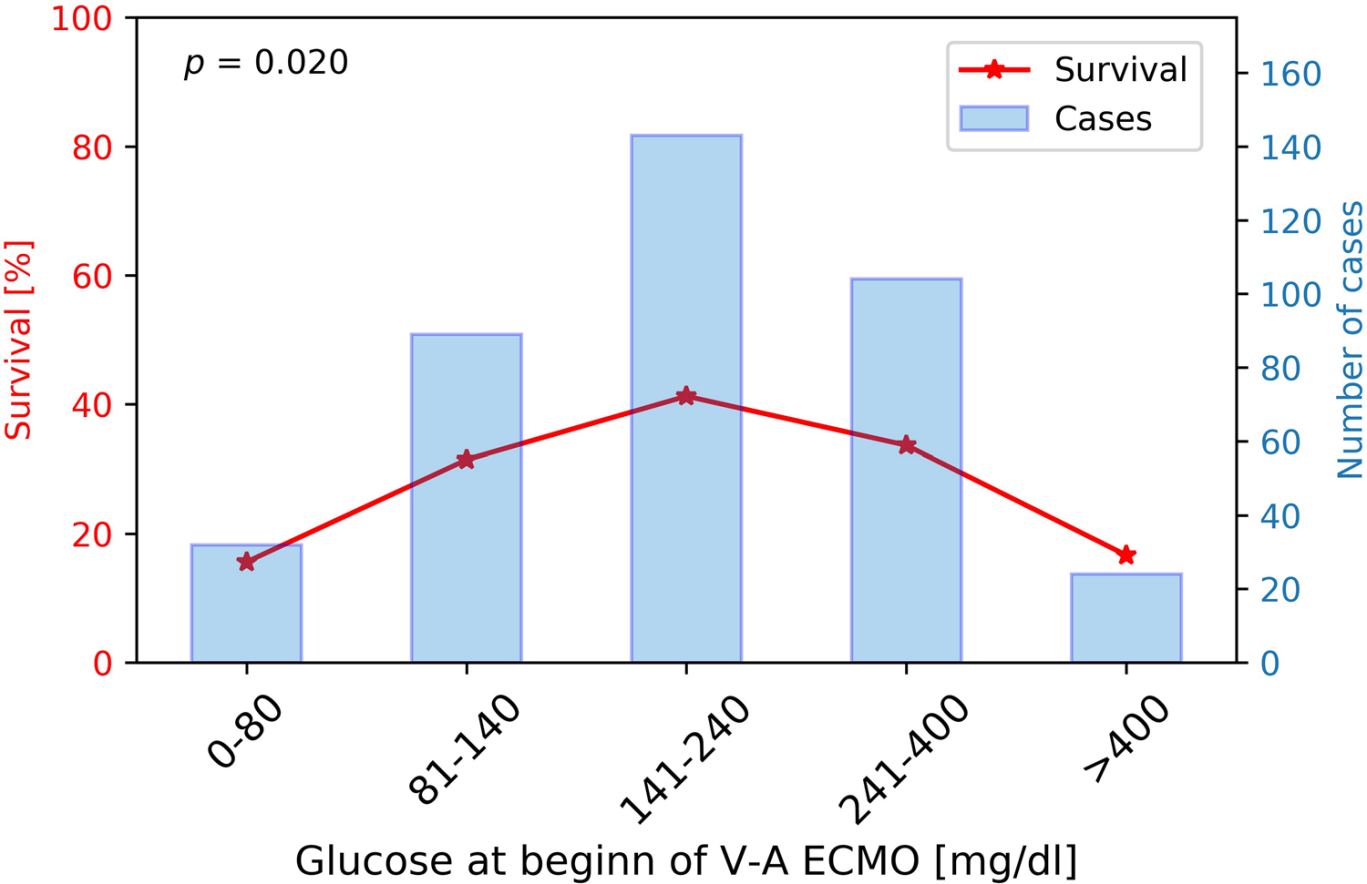
Survival in relation to different plasma glucose levels. The red stars represent the survival rate of each group, and the blue bars the number of patients per group

**Fig. 3:**
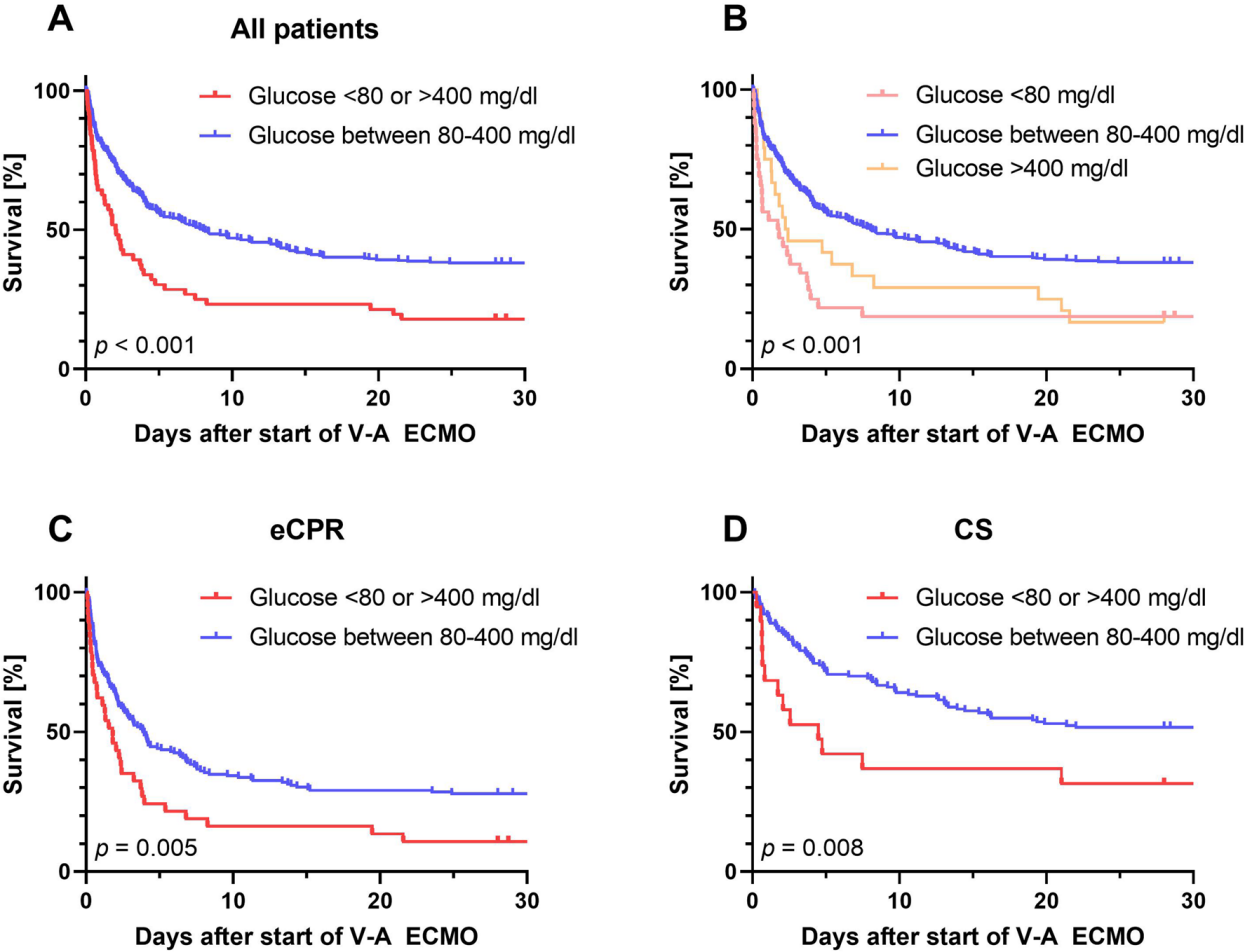
Kaplan–Meier survival curves. Glucose unit in mg/dl. **a** and **b** all patients with different grouping, **c** only ECPR patients, and **d** only patients with cardiogenic shock. *V-A ECMO* venoarterial extracorporeal membrane oxygenation therapy, *ECPR* extracorporeal cardiopulmonary resuscitation, *CS* cardiogenic shock

**Fig. 4:**
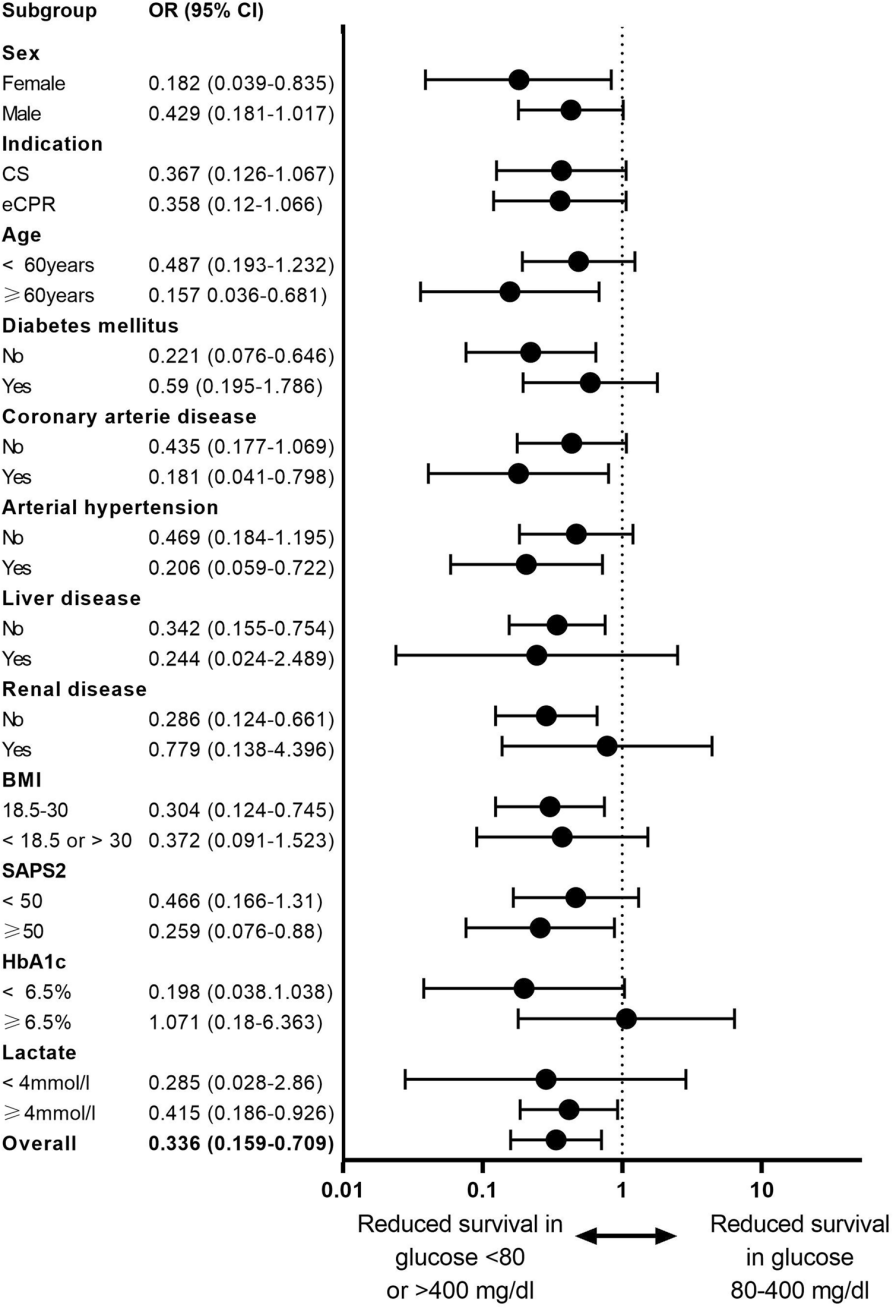
Forrest plot of different subgroups. *OR* Odds ratio, *CI* confidence interval, *ECPR* extracorporeal cardiopulmonary resuscitation, *CS* cardiogenic shock

Arterial glucose and outcome: 56/392 (14.3%) patients showed extreme arterial blood glucose levels either above 400 mg/dl or below 80 mg/dl at time of cannulation for V-A ECMO. We observed a significant association of hospital survival with glucose levels determined at start of V-A ECMO. Survival in patients with extreme glucose levels above 400 mg/dl or below 80 mg/dl was 16.1% compared to 36.3% in patients with arterial glucose of 80–400 mg/dl (*p* = 0.020, Fig. 2). Lower survival occurred in both groups, patients with blood glucose levels < 80 mg/dl and > 400 mg/dl (*p* < 0.001, Fig. 3). A subgroup analysis of patients with cardiogenic shock patients or ECPR revealed lower survival in patients with extreme glucose levels in both indications (*p* = 0.005 for ECPR and *p* = 0.008 for cardiogenic shock, Fig. 3). Additional subgroup analyses including sex, age, elevated HbA1c over 6.5%, and high lactate values over 4 mmol/l consistently revealed a trend towards reduced survival in the extreme glucose group in all subgroups except for HbA1c over 6.5% (Fig. 4).

Adjustment of confounders: In a multivariate logistic regression analysis, arterial glucose at V-A ECMO cannulation was an independent predictor of in-hospital mortality (*p* = 0.019, Table 3). Adjusting for significant predictors of hospital mortality detected in the multivariate logistic regression analysis in a Cox regression model confirmed that glucose is associated with poor outcome (*p* = 0.032, Table 4).

**Table 3:** Multivariate logistic regression analysis for hospital mortality

	OR	95% CI	*p* value
ECPR	2.410	1.50–3.87	< 0.001
Female	0.990	0.59–1.67	0.980
Diabetes mellitus	0.820	0.47–1.42	0.480
Age > = 60 years	1.620	0.99–2.67	0.060
Extreme glucose level	2.370	1.07–5.22	0.030
SAPS II > 50	2.480	1.52–4.04	< 0.001
Lactate > 4 mmol/l	1.800	1.02–3.17	0.040

*OR* Odds ratio, *CI* confidence interval

**Table 4:** Cox regression analysis for hospital mortality

	HR	95% CI	*p* value
ECPR	1.970	1.51–2.57	< 0.001
Extreme glucose level	1.760	1.27–2.44	< 0.001
SAPS II > 50	2.000	1.53–2.62	< 0.001
Lactate > 4 mmol/l	1.570	1.10–2.24	0.010

*OR* Odds ratio, *CI* confidence interval

## Discussion

We found a strong, inverse U-shaped association of arterial blood glucose at time of V-A ECMO cannulation and in-hospital survival. This correlation persisted in all investigated models and after adjustment for confounders.

Our findings add on previous studies reporting an association of dysglycemia with mortality. Indeed, in numerous reports from critically ill patient cohorts, hyperglycemia correlated with prognosis. Hyperglycemia is associated with adverse outcomes in critically ill patients with cardiac shock not treated with V-A ECMO [3], after myocardial infarction [15], pneumonia [16], or in mixed collectives of ICU patients [17]. This observation, however, is not new [18]. It has been known for over a century that patients with hemorrhagic shock can develop a hyperglycemia in response to tissue ischemia [19]. A similar correlation between hypoglycemia and poor outcome has been reported [20, 21]. This results in a U-shaped correlation with poor prognosis in dysglycemic patients after myocardial infarction [22]. While some studies suggest that after adjustment for blood lactate, stress hyperglycemia is not associated with outcome [23], other studies suggest hyperglycemia to be an independent predictor of unfavorable outcome [3].

In our study, we found a trend towards higher mortality for dysglycemic patients in all investigated subgroups, except for the diabetic subgroup. The latter is in line with previous studies, suggesting that the association of hyperglycemia with poor outcome is much weaker in diabetic patients [24] and supports the notion that acute rather than chronic hyperglycemia is associated with adverse outcomes [25]. The important question remains, however, in what way dysglycemia causes an increase in all-cause mortality. It has been suggested that hyperglycemia was proportional to the extent of injury [26] and might be a marker for endogenous stress.

There is an ongoing debate whether hypo- and hyperglycemia are only markers of disease severity or causally related to increased mortality and a potential therapeutic target [18]. While preventing hypoglycemia clearly improves outcome, a tight glucose control did not improve outcome in various randomized-controlled trials [27–29]. It has even been suggested that stress hyperglycemia is an essential survival response [30], a hypothesis that matches our data since best survival was detected in the patient group with moderately elevated blood glucose levels (140–240 mg/dl) at cannulation.

Extreme glucose levels have been associated with an increased risk for detrimental arrhythmia [31–33], cardiogenic shock [34], and cardiovascular death [33]. Importantly, we only investigated patients under V-A ECMO therapy, which should be the best possible therapy to compensate for arrhythmia or impaired tissue perfusion. The fact that the association between outcome and dysglycemia persists under V-A ECMO therapy therefore argues against a causal relationship. However, this should be evaluated in a prospective study. Our results nevertheless encourage incorporating arterial blood glucose in prognostication of patients after cannulation for V-A ECMO.

## Conclusion

Arterial blood glucose at time of V-A ECMO cannulation predicts in-hospital survival of patients with cardiac shock or after ECPR. Whether dysglycemia represents a potential therapeutic target requires further evaluation in prospective studies.

## Supplementary Information

Below is the link to the electronic supplementary material.

Supplementary file1 (DOCX 15 kb)

## Data Availability

The datasets used and analyzed during the current study are available from the corresponding author on reasonable request.

## Abbreviations

BE: Base excess
BMI: Body mass index
CAD: Coronary artery disease
CO: Carbon monoxide
CS: Cardiogenic shock
ECPR: Extracorporeal cardiopulmonary resuscitation
Hb: Hemoglobin
HbA1c: Glycated hemoglobin
HCO_3_: Bicarbonate
HCT: Hematocrit
ICU: Intensive-care unit
IHCA: In hospital cardiac arrest
OHCA: Out of hospital cardiac arrest
PAD: Peripheral artery disease
pCO_2_: Partial pressure of carbon dioxide
pO_2_: Partial pressure of oxygen
POCT: Point of care testing
ROSC: Return of spontaneous circulation
SAPS2: Simplified Acute Physiology Score 2
SBC: Standard bicarbonate concentration
SpO_2_: Peripheral oxygen saturation
V-A ECMO: Venoarterial extracorporeal membrane oxygenation therapy
