# Supplementary material for: Admission blood glucose level and outcome in patients requiring venoarterial extracorporeal membrane oxygenation
Source: Clin Res Cardiol. 2021 May 4;110(9):1484–92. doi: 10.1007/s00392-021-01862-7 (PMC8405505; doi:10.1007/s00392-021-01862-7)
Supplement: Supplementary file 1 — Supplementary file1 (DOCX 15 kb) [file 392_2021_1862_MOESM1_ESM.docx]

|  | All patients (n = 392) | Deceased (n = 261) | Survived (n = 131) | *p*-value |
| --- | --- | --- | --- | --- |
| Hospital survival | 131 (33.5%) |  |  |  |
| Female sex | 108 (27.6%) | 71 (27.2%) | 37 (28.2%) | 0.457 |
| Age [years] | 62.0 (51.5-70.0) | 63.0 (54.0-72.0) | 58.0 (47.0-68.0) | **0.008** |
| BMI [kg/m2] | 26.7 (24.2-27.7) | 26.7 (25.1-27.7) | 25.1 (24.1-28.7) | 0.502 |
| CAD | 216 (55.1%) | 145 (55.6%) | 71 (54.2%) | 0.840 |
| Arterial hypertension | 163 (41.6%) | 103 (39.7%) | 60 (45.8%) | 0.374 |
| PAD | 26 (6.6%) | 15 (5.8%) | 11 (8.4%) | 0.587 |
| Liver disease | 26 (6.6%) | 16 (6.1%) | 10 (7.63%) | 0.782 |
| Renal disease | 63 (16.1%) | 42 (16.1%) | 21 (16.0%) | 0.927 |
| Diabetes mellitus | 99 (25.3%) | 67 (25. 7%) | 32 (24.4%) | 1.00 |
| Lung disease | 62 (15.8%) | 38 (14. 6%) | 24 (18.3%) | 0.351 |
| SAPS II at admission | 54.0 (43.5-63.0) | 57.0 (48-65.0) | 46.0 (37.0-55.0) | **<0.001** |
| Duration of ICU stay [days] | 5.0 (1.5-13.9) | 2.4 (0.7-6.4) | 16.0 (9.0-27.0) | **<0.001** |
| V-A ECMO duration [hours] | 68.6 (26.3-119.7) | 52.22 (16.82-107.4) | 87.33 (62.4-137.3) | **<0.001** |
| Indication for V-A ECMO |  |  |  | **<0.001** |
| Cardiogenic shock | 173 (44.1%) | 92 (35.3%) | 81 (61.8%) |  |
| ECPR | 219 (55.9%) | 169 (64.8%) | 50 (38.2%) |  |

Supplemental table 1 Patients characteristics and path of ICU stay separated by deceased vs. survived

Footnote Suppl. Table 1: BMI = Body mass index, CAD = Coronary artery disease, PAD = Peripheral artery disease, SAPS2 = Simplified Acute Physiology Score 2, ICU = Intensive care unit, V-A ECMO = venoarterial extracorporeal membrane oxygenation, ECPR = extracorporeal cardiopulmonary resuscitation.

|  | All patients (n = 392) | Deceased (n = 261) | Survived (n = 131) | *p*-value |
| --- | --- | --- | --- | --- |
| HbA1c [%] | 5.8 (5.3-6.4) | 6.05 (5.55-6.7) | 5.7 (5.2-6.1) | **0.003** |
| Glucose [mg/dl] | 184.5 (122.5-272) | 186 (117-280) | 182 (139-264) | 0.677 |
| pH | 7.27 (7.17-7.35) | 7.24 (7.14-7.33) | 7.3 (7.22-7.38) | **<0.001** |
| pCO2 [mmHg] | 38.75 (32.7-45.6) | 38.8 (33.4-46.4) | 38.7 (30.1-43.9) | **0.044** |
| pO2 [mmHg] | 184 (102-344) | 197.5 (98.6-374) | 168 (108-292) | 0.259 |
| SpO2 [%] | 99 (97-99.7) | 98.9 (96.5-99.7) | 99.1 (97.6-99.7) | 0.189 |
| HCO3 [mmol/l] | 17.2 (13.7-20.7) | 16.3 (13-20) | 18.9 (15.65-22.05) | **0.001** |
| SBC | 17.4 (13.55-20.95) | 16.3 (12.8-20) | 18.8 (15.4-21.55) | <**0.001** |
| Hb [g/dl] | 10.1 (8.2-12.2) | 9.8 (7.7-11.95) | 10.8 (9.1-12.9) | **<0.001** |
| HCT [%] | 30.6 (25.1-36.8) | 29.6 (23.2-35.3) | 32.7 (27.6-39.7) | **<0.001** |
| Sodium [mmol/l] | 139.2 (135-143) | 139.8 (136-143) | 139 (134.5-142) | 0.316 |
| Potassium [mmol/l] | 4.3 (3.74-4.96) | 4.4 (3.8-5.1) | 4.11 (3.7-4.7) | **0.009** |
| Chloride [mmol/l] | 106 (102-110) | 105 (101.5-109) | 107 (103-111) | 0.016 |
| Calcium [mmol/l] | 1.08 (1.01-1.15) | 1.06 (0.99-1.14) | 1.1 (1.05-1.17) | **<0.001** |
| Lactate [mmol/l] | 9.55 (4.94-13.4) | 10.9 (6.8-14.49) | 6.61 (3.2-10.23) | **<0.001** |
| Bilirubine [mg/dl] | 0.8 (0.5-1.4) | 0.9 (0.5-1.5) | 0.7 (0.4-1.4) | 0.311 |

Supplemental table 2 Laboratory and POCT values at time of cannulation for V-A ECMO separated by deceased vs. survived

Footnote Suppl. Table 2: pCO2 = partial pressure of carbon dioxide, pO2 = partial pressure of oxygen, SpO2 = peripheral oxygen saturation, Hb = Hemoglobin, HCO3 = Bicarbonate, HCT = Hematocrit, SBC = Standard bicarbonate concentration.
